# The Influence of the Surface Chemistry of Cellulose Nanocrystals on Ethyl Lauroyl Arginate Foam Stability

**DOI:** 10.3390/polym14245402

**Published:** 2022-12-09

**Authors:** Agnieszka Czakaj, Emmanouil Chatzigiannakis, Jan Vermant, Marcel Krzan, Piotr Warszyński

**Affiliations:** 1 Jerzy Haber Institute of Catalysis and Surface Chemistry, Polish Academy of Sciences, ul. Niezapominajek 8, 30-239 Krakow, Poland; 2 Polymer Technology Group, Department of Mechanical Engineering, Eindhoven University of Technology, P.O. Box 513, 5600 MB Eindhoven, The Netherlands; 3 Department of Materials, ETH Zürich, Vladimir-Prelog-Weg 5, 8093 Zürich, Switzerland

**Keywords:** foam stability, dynamic thin-film balance, cellulose nanocrystals, surface chemistry, surface tension, fluid film, surface dynamics, interfacial phenomena

## Abstract

Guanidine-based surfactant ethyl lauroyl arginate (LAE) and cellulose nanocrystals (CNCs) form complexes of enhanced surface activity when compared to pure surfactants. The LAE-CNC mixtures show enhanced foaming properties. The dynamic thin-film balance technique (DTFB) was used to study the morphology, drainage and rupture of LAE-CNC thin liquid films under constant driving pressure. A total of three concentrations of surfactant and the corresponding mixtures of LAE with sulfated (sCNC) and carboxylated (cCNC) cellulose nanocrystals were studied. The sCNC and cCNC suspension with LAE formed thin films, with stability increasing with surfactant concentration and with complex rheological properties. In the presence of LAE, the aggregation of CNC was observed. While the sCNC aggregates were preferentially present in the film volume with a small fraction at the surface, the cCNC aggregates, due to their higher hydrophobicity, were preferentially located at film interfaces, forming compact layers. The presence of both types of aggregates decreased the stability of the thin liquid film compared to the one for the LAE solution with the same concentration. The addition of CNC to LAE was critical for foam formation, and foam stability was in qualitative agreement with the thin films’ lifetimes. The foam volume increased with the LAE concentration. However, there was an optimum surfactant concentration to achieve stable foam. In particular, the very resistant foam was obtained with cCNC suspensions that formed the interfaces with a complex structure and rheology. On the other hand, at high LAE concentrations, the aggregates of CNC may exhibit antifoaming properties

## 1. Introduction

Foamability and foam stability are interesting for many technological processes, including flotation, cleaning, cosmetic application and food processing. Immediately after its formation, foam undergoes various simultaneous and inter-related disruptive processes such as coarsening, drainage and coalescence. Foams with prolonged stability can be created from dispersions of surfactant with nanoparticles or even from surface active particles without surfactants [1]. Mixed systems allow us to obtain the desired technological properties with the reduced amount of surfactant, and thus, with lower costs and environmental impact. Foam can be treated as the ensemble of connected bubbles separated by thin liquid films of a continuous liquid phase [2]. Apart from the adsorption and desorption of surface-active species and their effect on surface tension, many other factors such as capillarity, hydrodynamic forces, interfacial rheology and intermolecular interactions play a role in thin liquid film stability. In particular, the film’s critical thickness, at which its rupture is observed, increases with the applied pressure drop [3]. Minor differences in the balance of forces can result in thin films’ lifetimes spanning over six orders of magnitude [3]. 

Recent progress in film dynamics has been facilitated by the development of the dynamic thin-film balance (DTFB) technique [4] with improved pressure and temperature control, which originated from the setup of Sheludko [5], and the single bubble with the interferometric technique for precise control of spatially resolved film thickness. In a modified version of the Sheludko cell, which consists of a microfabricated thin-film holder that resembles a bike wheel, the thin-film hole is connected radially to the external annulus by 24 channels. Therefore, larger disjoining pressures can be measured, the drainage is radial and symmetric, and the device is suitable for a small number of samples and reuses [4,6,7,8,9,10,11].

The generalised Stokes–Laplace–Reynolds equation describes the dynamic pressure balance that characterises the drainage of the thin liquid film [4]. The hydrodynamic pressure *P_H_*(*h*, *r*) is given by a local pressure balance:

(1) $$P_{c}+\frac{2\sigma }{R}=P_{H}\left(h,r\right)+P_{\infty }-\prod_{d}\left(h,r\right)+\frac{\sigma }{2r}\frac{\partial }{\partial r}\left(r\frac{\partial h}{\partial r}\right)$$

where *P_c_* is the externally applied pressure across the film, which induces drainage, 2*σ*/*R* is the Laplace pressure due to curvature of the Plateau border (*σ* is the surface tension and *R* is the bike wheel hole’s radius), and *P_∞_* is the pressure at the meniscus, *∏_d_*(*h*,*r*) is the disjoining pressure; the last term describes local Laplace contributions of curvature differences [4].

In general, the equilibrium properties, such as the disjoining pressure, do not suffice to explain the thinning and rupture dynamics, and hydrodynamics need to be studied using the DTFB. It can give complementary information to other experimental surface science techniques such as interfacial rheology. In particular, dynamic thin-film balance enables us to visualise surface flows and assess whether interfaces are stress-carrying (immobile) or stress-free (mobile), and hence provide insight into the effect of Marangoni stresses or surface rheology. Moreover, with the DTFB technique, one can visualise particles structuring in the thin film, the existence of the aggregates, nucleation of lipids and black film formation [4]. 

Ethyl lauroyl arginate is an arginine-based, biodegradable surfactant with strong surface activity. Combined with cellulose nanocrystals, it allows the formation of stable, environmentally friendly foams. In previous work, some of the present authors used the dynamic fluid interferometry/rising bubble technique. They observed significant immobilisation of the interface in thin films generated from solutions of ethyl lauroyl arginate (LAE) and cellulose nanocrystals (sCNCs) with sulfate hydrophilic groups [12]. A significant immobilisation of the interface in thin films was revealed. The drainage of an initially dimpled thin film of the mixture of surfactant with non-surface-active nanoparticles was significantly slower compared to the pure surfactant solution with the equivalent concentration. Within the narrow concentration range that was studied, film stability was found to depend on the surfactant/nanoparticle ratio. The intermediate surfactant concentration, mixed with CNCs and filtered, exhibited the highest coalescence time. The drainage of non-filtered LAE-CNC solution was even slower and proceeded by a dynamic reorganisation of large cellulose nanocrystal aggregates, which in other cases usually act as antifoam species. The LAE-CNC dispersions had high interfacial shear elasticity. Other researchers found a comparable value of interfacial shear elasticity of the same concentration (0.3% by weight) of CNCs in solutions where electrostatic repulsive interactions were screened by adding salt [13]. The possible effect of bulk viscosity in such concentrations of high-aspect-ratio CNCs cannot be excluded.

The CNCs are highly charged with a predominantly hydrophilic surface; thus, a hydrophobic modification is needed for their attachment to the air/water interface [14]. Undoubtedly, a CNC suspension without the addition of a surfactant does not foam. Thus, the hydrophobic modification of CNCs is standard practice. One such example is the hydrophobic modification of cellulose in the form of nanofibers and nanocrystals (length 300 nm) by adsorption of cationic octylamine, which enhances foam stability [15]. The improved foam stability was attributed to the increased bulk viscoelastic properties and nanocellulose charge. Positively charged ethyl lauroyl arginate interacts electrostatically with negatively charged cellulose nanocrystals, modifies their surface properties and induces aggregation. With both hydrophobic modification and aggregation at play, it is hard to predict the surface tension of surfactant–nanoparticle mixtures. For example, didecyldimethyl ammonium bromide had lower surface activity in CNC suspensions than pure surfactant [16]. On the contrary, the surface activity of commercial LAE in the mixture with CNCs was prevalently higher than pure surfactant [12,13]. The rate of addition of a surfactant may change the aggregation path of nanoparticles; hence, it has consequences for the hydrodynamic diameter of the dispersion and its turbidity [12,13]. For the CNC suspensions and surfactant concentration c≪CMC, the zeta potential of cellulose nanocrystals does not significantly change. For example, adding LAE at concentrations below 0.01 wt%. neither decreases the negative value of CNCs’ zeta potential nor increases their hydrodynamic diameter [12,13].

The solution of ethyl lauroyl arginate usually contains other surface-active substances, impurities from synthesis and hydrolysis products that modify the kinetics and equilibrium surface tension of solutions. As described before [17], fresh LAE solution hydrolyses to Nα-lauroyl–L-arginine (LAS) or dodecanoic acid, which may form the heterodimers LAE-dodecanoate anion or LAE-LAS. The surface activity of such mixed solutions is significantly changed, and the complexity of the surfactant’s rheological response increases. Thus, in the mixture with cellulose nanocrystals, various surface-active compounds exist with different interactions with cellulose nanocrystals. Additionally, the base-catalysed hydrolysis of LAE induces a decrease in the solution pH that may influence the weakly charged groups at the surface of the cellulose nanoparticles.

In this work, thin-film balance drainage experiments coupled with micro-interferometric imaging were carried out under an applied constant pressure drop. The experiment was designed to simulate pressure changes accompanying bubbles prior to coalescence and compare cellulose nanocrystals’ effect with different surface hydrophilic groups with respect to foaming properties. Different particles were chosen because the aggregation ratio varies the rheological response and surface flow to a great extent [18,19,20]. Our work aims to fill a gap in nanocellulose research concerning the effect of CNCs’ surface chemistry on their application potential. Although researchers point out that cCNCs find applicability in many technologies, their interfacial properties have not been studied, specifically for foaming. Moreover, to the best of our knowledge, the thin-film balance technique has not been used so far to study the effect of surfactants and cellulose nanocrystals on thin film behaviour. 

Analytical grade ethyl lauroyl arginate and two types of commercially available cellulose nanocrystals were used: sCNCs with sulfate ester groups and cCNCs with carboxyl groups, both having comparable size and surface charge [12,13]. Industrially produced CNCs compare well with CNCs extracted at a bench scale, with all material containing highly crystalline, high-aspect-ratio cellulose nanocrystals [21,22,23,24,25]. Sulfate-based cellulose nanocrystals derived from cellulose by sulfuric acid hydrolysis are widely available. Carboxylic cellulose nanocrystals are manufactured with the technology of H_2_O_2_ oxidation [21,22]. So far, they have not been well characterised yet for their interfacial properties. Both sulfate ester and carboxylic CNCs are particles with faces of different hydrophilicity, and tuning their exposure to water might be essential for foaming properties [12,13,26]. By combining the TFB technique and interfacial properties measurements, we show how the complex interactions between the surfactant and the CNCs affect the surface pressure, disjoining pressure, surface rheology and adsorption dynamics in mixed LAE-CNC systems.

## 2. Materials and Methods

Ethyl lauroyl arginate, United States Pharmacopeia analytical standard (declared purity of 99%), was purchased from Merck. The stock solution was prepared in cold deionised water (4 ℃, 20 MΩ cm) and then diluted to the appropriate concentration. Stock solution and dilutions were used within one day if not described otherwise. Cellulose nanocrystals with sulfate half ester groups were purchased from Celluforce (diameter 5 nm, length 100 nm, sulfate content 0.25 mmol/g). Cellulose nanocrystals with carboxyl groups were purchased from Anomera (zeta potential range −40 to −50 mV, diameter 5–10 nm, length 150–200 nm, carboxyl content 0.12–0.20 mmol/g). Cellulose nanocrystals were dispersed carefully in water to achieve a concentration of 0.6% by weight and were subsequently sonicated. The CNC solution was added dropwise to the surfactant solution under constant stirring to achieve a final concentration of 0.3 wt% cellulose nanocrystals in all solutions.

### 2.1. Surface Activity and Dilation Rheology

The surface tension of samples was measured immediately after surfactant solution or dispersion preparation using the pendant drop technique with a Sinterface PAT-1M tensiometer (Sinterface Technologies e.K., Berlin, Germany). A drop of solution (11 µL) was created from a 2 mm diameter capillary and kept in the thermostated chamber for up to 2000 s. The drop profile was monitored and fitted with the Young–Laplace equation to calculate the surface tension until it did not change during the consecutive measurements. Then, the value of the equilibrium surface tension was recorded. 

For the dilational rheological measurements, drop-size oscillations were applied after reaching the surface tension equilibrium by imposing drop volume (area) changes of less than 10% of the volume. Fourier transform of the surface tension variations was calculated, and an apparent surface dilational modulus was determined as a complex number. 

### 2.2. Particle Characterisation

The size of CNC nanoparticles was measured by dynamic light scattering with the Malvern Nano ZS instrument (Malvern, Worcestershire, UK). Each measurement was repeated three times. The zeta potential of all LAE-CNC suspensions was measured by laser Doppler velocimetry with the Malvern Nano ZS instrument. Each measurement was repeated three times. No viscosity correction was applied. The average error (standard deviation) was 5 mV maximum.

### 2.3. Thin-Film Balance

**Figure 1:**
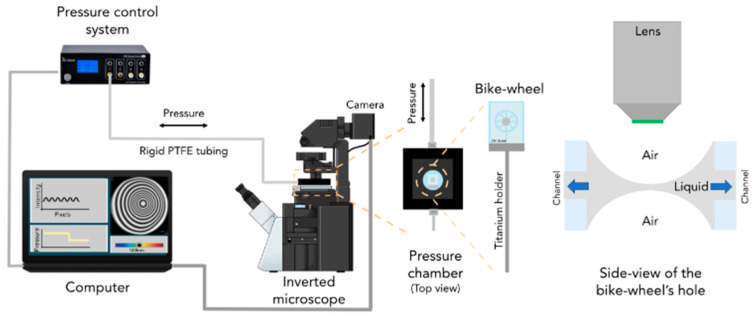
Scheme of the thin-film balance setup used in the film imaging experiments. Reprinted from [4].

The setup of the TFB, as illustrated in Figure 1, and the experimental procedure were described previously by Chatzigiannakis et al. [4]. It consists of an upright fixed-stage microscope, a pressure control system, an in-house fabricated anodised aluminium pressure chamber, in which the bike wheel microfluidic device was placed. The bike wheel chip was manufactured by photolithography. It consists of a diamond-drilled hole with a diameter of 1 mm and a thickness of 400 µm and 24 channels—the spokes of the bike wheel (width of 45 µm and depth of 20 µm) connected to the hole, all leading to a circular channel of larger dimensions. The chip was glued onto a titanium holder using two-component epoxy. The pressure was controlled by a piezoelectric pressure control system with a resolution of 1 Pa and a maximum pressure of 20 kPa. It was connected to the pressure chamber by rigid PTFE tubing with an inner diameter of 0.1 mm. The film was visualised with a Nikon Eclipse FN1 fixed-stage upright microscope (to minimise vibrations) and a 10x long working distance objective with a mounted Hamamatsu ORCA-Flash4.0 CMOS camera (Hamamatsu Photonics Europe GmbH, Herrsching, Germany). A monochromatic wavelength of 508 nm was used for the reflection. Its digitalised image was converted to thickness according to the Sheludko equation [4,5].

The experimental procedure consisted of the following steps. Initially, the thick fluid film (micron-size thickness) was created in the orifice of the thin-film balance. By adjusting the pressure in steps of 1 Pa, the equilibrium pressure *P_eq_* was determined and the film was visible when the first interference fringes appeared at the thickness of a few μm. *P_eq_* is the sum of all the contributions in the static thick film (cf. Equation (1)). After that, the pressure inside the film was increased with the applied pressure step of 100 Pa. The film started to drain. Buildup of hydrodynamic pressure caused the expansion of the film. A sequence of images was collected by the camera with a maximum 10 ms temporal resolution. The measured coalescence time (film lifetime) corresponded to the time interval between the onset of film expansion and the rupture of the film. 

### 2.4. Foaming

The double-syringe method was applied for foaming experiments. Foams were generated by manually pushing 15 mL LAE-CNC solution and 30 mL of air from one medical-grade syringe to the other syringe connected to the former through a narrow tube [12,13,27,28,29]. After ten cycles, syringes were left in the vertical position. Initial foam volume was ascribed as the volume after 1 min from the foam formation. 

## 3. Results and Discussion

### 3.1. Suspension Characterisation

The surface tension of analytical standard LAE and the suspension of nanoparticles with LAE are given in Table 1.

Depending on LAE concentration, the equilibrium surface tension of the suspension is either lower, similar or higher than the surface tension of the surfactant solutions. At low LAE concentrations, the nanoparticles are partly hydrophobised, and the synergistic effect of LAE and CNC on surface activity was observed. This effect is stronger for the cCNC suspension. At high LAE concentrations, the surface activity is surfactant-dominated, but some surfactant is consumed by adsorption at nanoparticles. All studied LAE concentrations were much smaller than critical micelle concentration CMC, which for the surfactant analytical standard surfactant was 1.0 mM [12,13,17].

The hydrodynamic diameter and zeta potential of the cellulose nanoparticle suspension, as well as a suspension with LAE surfactant, are given in Table 2.

The size of cCNCs seems to be systematically smaller and less monodisperse than sCNCs, while their zeta potential is less negative, significantly decreasing with the addition of LAE. That could be the result of decreasing the negative charge of cCNCs by LAE adsorption; however, that effect seems to be absent for the sCNC suspension. On the other hand, it may be due to a partial protonation of surface carboxyl groups as the pH of the suspension decreases due to the hydrolysis of LAE.

The concentration of CNCs (0.3 wt%) was selected to minimise the influence of the bulk viscosity on the interfacial properties of the suspensions and, possibly, on the foam stability. It was shown that, below the concentration of 1%, CNC viscosity is only slightly modified, and suspensions do not show shear-thinning behaviour [26]. The viscosity for the sCNC and LAE-sCNC suspensions in the studied concentration range was determined previously by some of the authors [12,13]. The measured viscosity of 1.3 mPas for sCNC CelluForce, in agreement with the literature [21,22,23,24,25,26,30], was the same for cellulose nanocrystals and LAE-sCNC (at 0.23 mM LAE). Moreover, the estimated rate of the film thinning was the same for 0.35 mM and 1 mM of LAE and 0.3 wt% of sCNCs. Knowing the rate of the thin-film thinning is directly dependent on viscosity, we concluded that minor viscosity differences were not relevant for LAE-CNC film lifetimes and foam stability. A small viscosity increase to 1.6 mPa s was observed for 0.35 mM LAE and sCNCs, which was attributed to the aggregation confirmed by DLS measurements [12,13]. Therefore, that concentration was chosen as the maximum concentration for further experiments. We assumed that, up to that concentration, a similar viscosity for cCNCs was expected. As was demonstrated by Delepierre et al. [30], the viscosity of 2wt% cCNCs, which was a concentration over three times higher than in our case, was only 1.6 mPa s (10 s^−1^).

### 3.2. Thin-Film Balance

The films of the pure LAE surfactant at a concentration of 0.075 M drained fast down to an equilibrium thickness of approximately 15 nm. Drainage occurred in 5–10 s, depending on surfactant concentration, and proceeded symmetrically. The short drainage times are expected for these concentrations in surfactant films [31,32] and are indicative of the relatively small magnitude of Marangoni stresses opposing the bulk outflow of the liquid. Drainage stopped when the equilibrium thickness was reached. This thickness resulted from the repulsive electrostatic interactions counteracting the sum of the applied pressure and the attractive van der Waals forces.

**Figure 2:**
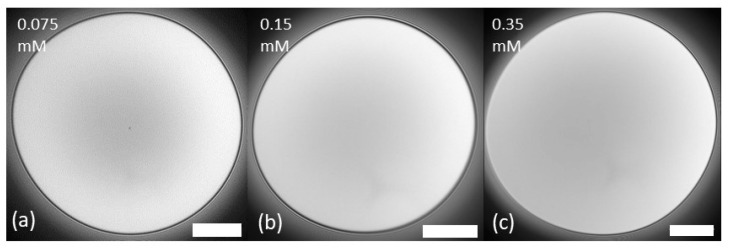
Morphology of LAE surfactant films after the pressure step of 100 Pa at the end of the drainage. (**a**) LAE 0.075 mM (the film was unstable, the image was taken before the rupture after c.a. 40 s drainage); (**b**) LAE 0.15 mM; (**c**) LAE 0.35 mM. Scale bar 100 μm.

The film with 0.075 mM LAE was already unstable at a 100 Pa pressure change, while those with 0.15 mM LAE and 0.35 mM LAE were stable at this initially applied pressure. Images of films at the end of drainage after the applied 100 Pa pressure step are presented in Figure 2. Additional pressure ramps for the assessment of the critical pressure for rupture revealed that film with 0.15 mM LAE broke at the applied pressure of 200 Pa, whereas 0.35 mM LAE broke at 1250 Pa. That can be attributed to the increase in the electrostatic disjoining pressure. With increasing LAE concentration, its adsorption at water/air interface [33] and, consequently, the interfacial charge increases. The same phenomenon was observed for the solution of another cationic surfactant, tetradecyl trimethyl ammonium bromide [33]. 

In pure surfactant solutions, below critical micelle concentration that form common black films of almost constant thickness morphology, the differences are usually not observed. However, for the lowest concentration, a small black spot in the middle of the film can be seen. The small spot is attributed to limited surfactant spreading at low concentrations [34].

**Figure 3:**
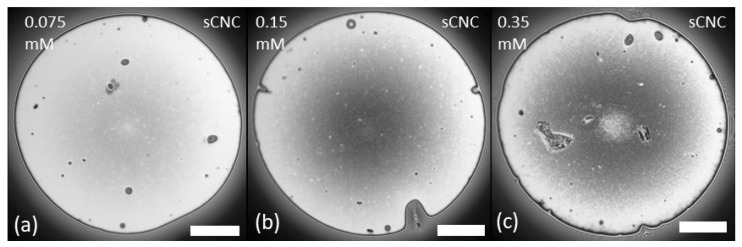
Morphology of LAE-sCNC films at the end of drainage after the pressure step of 100 Pa. (**a**) CNC-LAE 0.075 mM; (**b**) CNC-LAE 0.15 mM; (**c**) CNC-LAE 0.35 mM. Cellulose nanocrystal concentration, 0.3 wt%. Scale bar is 100 μm.

**Figure 4:**
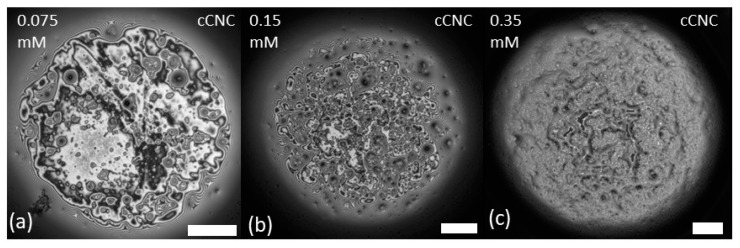
Morphology of LAE-cCNC films after the pressure step of 100 Pa at the end of the drainage. (**a**) CNC-LAE 0.075 mM; (**b**) CNC-LAE 0.15 mM; (**c**) CNC-LAE 0.35 mM. Cellulose nanocrystal concentration, 0.3 wt%. Scale bar is 100 μm.

The morphology of mixed LAE-CNC thin films was more complex and, as illustrated in Figure 3 and Figure 4, dependent on the ratio of surfactant/particle concentration. The morphology and drainage of films depended on the type of CNC: sulfated or carboxylated. Drainage is presented as videos in the Supplementary Materials: Video S1: LAE 0.075 mM, Video S2: LAE 0.15 mM, Video S3: LAE 0.35 mM, Video S4: LAE 0.075 mM sCNC, Video S5: LAE 0.15 mM sCNC, Video S6: LAE 0.35 mM SCNC, Video S7: LAE 0.075 mM cCNC, Video S8: LAE 0.15 mM cCNC, Video S9: LAE 0.35 mM cCNC.

**Figure 5:**
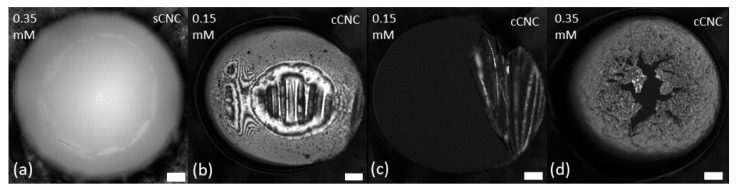
Morphology of LAE-cCNC films after film reforming. Complex interactions of LAE-sCNC (**a**), cCNC-LAE 0.15 mM while reforming the film after rupture (**b**,**c**) and cCNC-LAE 0.35 mM (**d**). Scale bar 100 μm.

As illustrated in Figure 3, all LAE-sCNC films at the end of drainage after the pressure step of 100 Pa were stable and relatively uniform, with most nanoparticles in the film volume and some nanoparticle aggregates at the interface. The film with sCNC-LAE 0.35 mM reached a steady state, and after 10 min, an additional 50 Pa pressure jump was applied. That resulted in the film breaking. After reforming the film with a new portion of the suspension, its morphology seemed to be uniform, with a small dimple (cf. Figure 5a). The coalescence time in a thin film for the medium concentration of LAE correlates well with results previously obtained for the moving bubble (DFI method) [12]. 

In contrast, as illustrated in Figure 4, the LAE-cCNC films were populated by larger cCNC aggregates for all surfactant concentrations. They seem to have the tendency to occupy the interface and the coverage increased with surfactant concentration. Film drainage occurred at regions where no aggregates were present, and its rupture was preceded by the formation of a Newton black film (regions with a thickness of approximately 10 nm), with the thin regions displacing the adsorbed particles. Such an effect hints that excess surfactant might compete for the surface, destabilising the CNC network.

When the film was reformed after rupturing, we observed the formation of a thick rigid interface formed with LAE-cCNC aggregates. The applied pressure change resulted in the wrinkling of the interfacial layer and, finally, the film’s rupture (cf. Figure 5b,c). For cCNC-LAE 0.35 mM, the thin film was much thicker, the structure less uniform, and the rupture proceeded with a surfactant black film displacing the cCNC-rich interface rather than wrinkling and folding (Figure 5d).

The observed differences between the film behaviour of sCNC and cCNC suspensions can originate from the more efficient hydrophobisation of cCNCs by LAE and their flow-induced aggregation during the film formation. Besides the electrostatic interaction, the guanidine group of LAE can form a bidentate hydrogen bond with the carboxyl surface group of cCNCs that contributes to enhanced hydrophobisation. 

**Figure 6:**
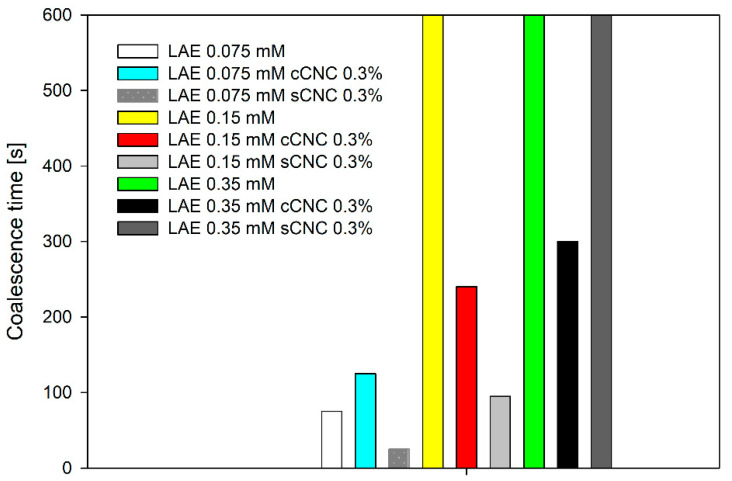
The thin films’ lifetime after equilibration and the pressure step of 100 Pa. For pure LAE, 0.15 mM and 0.35 mM LAE and 0.35 mM LAE and sCNC, film lifetime exceeded 600 s.

Film lifetime (coalescence time), which includes both drainage and rupture time, increased systematically with surfactant concentration without and for both particle types, as presented in Figure 6. As discussed above, the effect of higher viscosity can be excluded in the CNC concentration used in our experiments. The effect of the bulk viscosity for polymer solutions on coalescence time was characterised with dynamic thin-film balance, including the same pressure step of 100 Pa, as in this experiment [7]. The drainage time increased linearly with viscosity in agreement with the Stokes–Laplace–Reynolds equation for polymer solutions of polyisobutylene. The difference in coalescence time for solutions with viscosities of 3.5 mPa s and 7.5 mPa s was equal to 40 s. It cannot be expected that viscosity differences not exceeding 0.5 mPa s for LAE-CNC solutions (viscosities between 1.3–1.6 mPa s) can result in coalescence time differences reaching 50 s. The film coalescence time exceeded 600 s for LAE concentrations of 0.15 mM and 0.35 mM. The film lifetime of the suspension of CNC with LAE was, in general, shorter than that of the surfactant solution with the same concentration. That means that the presence of aggregates, either in the film volume (sCNCs) or at its surface (cCNCs), leads to its destabilisation. That can be explained by the antifoam action of large aggregates, which seems to be in agreement with our previous findings for the sCNCs and industrial-grade LAE [12,13] Alternatively, it can be explained by the decrease in the effective LAE concentration by complexation with CNCs and thus the decrease in the electrostatic disjoining pressure. On the other hand, the sCNC-LAE 0.35 mM film was very stable up to high pressure, which may result from the formation of a rigid, solid-like interface.

### 3.3. Interfacial Rheology

**Figure 7:**
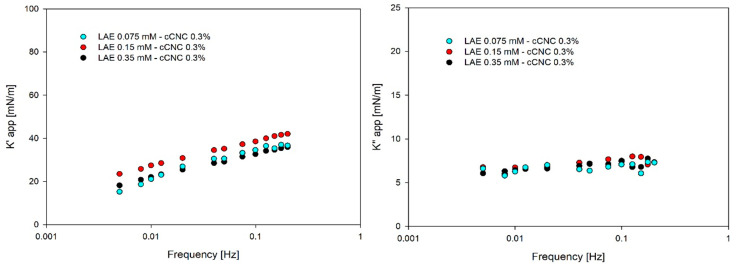
Apparent elastic (K′) and loss (K″) moduli for cCNC suspensions with various concentrations of LAE (0.075 mM, 0.15 mM and 0.35 mM).

**Figure 8:**
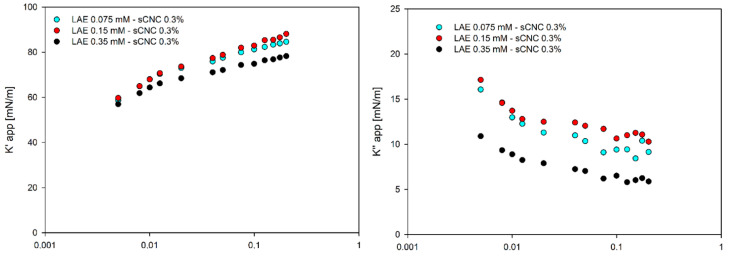
Apparent elastic (K′) and loss (K″) moduli for sCNC suspensions with various concentrations of LAE (0.075 mM, 0.15 mM and 0.35 mM).

The apparent elastic modulus in the mixture with cCNCs did not differ significantly from the values obtained for pure surfactant [12,13], and the apparent loss modulus was almost invariant to particle addition and concentration, as is illustrated in Figure 7. That indicates that the transfer of surfactant/nanoparticles is much faster than the drop oscillations. On the contrary, the apparent elastic modulus for sCNCs was almost three times higher than for cCNCs. In contrast, the apparent loss moduli for sCNCs decreased significantly with oscillation frequency, as shown in Figure 8. A high elastic modulus and the apparent decrease in the loss modulus are typically observed for rheological complex interfaces due to shear effects. These results, with high apparent elasticity for all LAE concentrations, seem to contradict the ones obtained with the thin-film balance technique, where LAE-CNC films were unstable for 0.075 mM LAE; however, one needs to consider different geometry and flow patterns that can favour nanoparticle aggregation in the thin fluid film. Furthermore, for rheologically active interfaces with cellulose nanocrystals, the drop shape might show deviations from the Young–Laplace equation [35,36]. Thus, the results of the pendant drop oscillations experiment, suitable for the determination of surfactant transport effects, cannot explain the complexity of the LAE-CNC interface and does not reflect the rheological response at the complex interface.

### 3.4. Foaming

**Figure 9:**
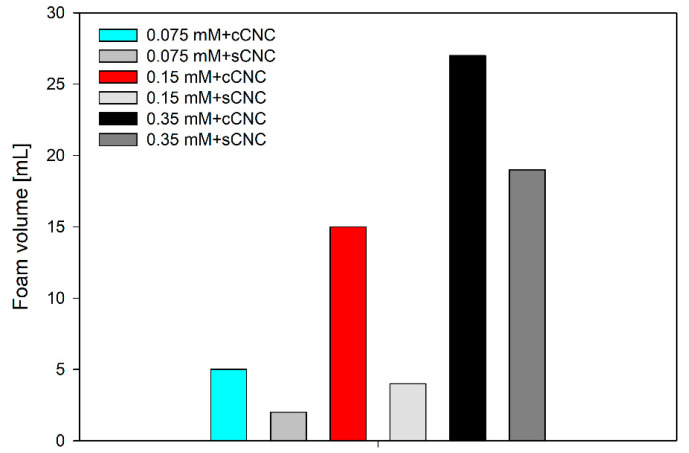
Initial foam volume obtained from 15 mL of LAE-cCNC or sCNC dispersed with double-syringe method. Foam volume was recorded 1 min after the foaming cycle.

**Figure 10:**
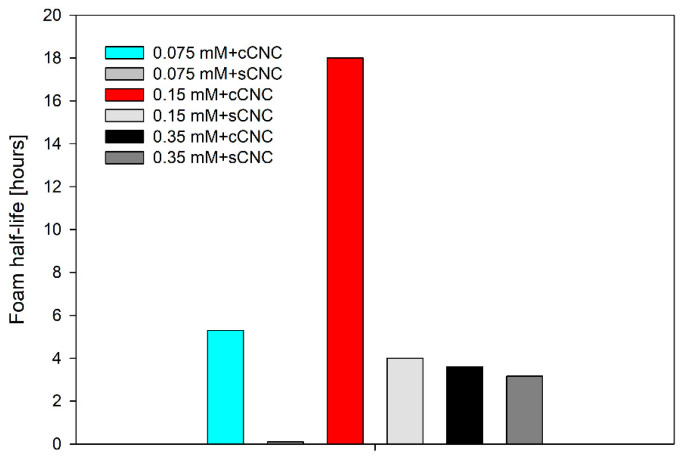
The half-life of foams formed in the CNC-LAE suspensions with the double-syringe method.

The results concerning the initial foam volume and foam half-life for suspensions of cellulose nanocrystals with LAE are presented in Figure 9 and Figure 10. For the selected LAE concentrations of 0.075 mM, 0.15 mM and 0.35 mM, it was impossible to produce stable foams with the double-syringe method. Apparently, the concentration of surfactant was too small to stabilise the foam, with a significant coarsening effect destabilising submillimiter bubbles. Mikhailovsakaya et al. [37] showed that the stability of thin films could be correlated directly if we study foam films at the same capillary number and limit coarsening effect. Relatively stable thin film obtained for 0.15 mM and 0.35 mM LAE concentrations in the thin-film balance experiment is only the estimate of the macroscopic system with the evolution of bubble size, non-uniform surface stresses and the time-dependent aggregation of nanoparticles.

In the suspension of cellulose nanocrystals with ethyl lauroyl arginate, the foamability was directly correlated to surfactant concentration. The same trend was observed in the thin films’ lifetime; however, the initial foam volume was c.a. two times higher for cCNCs than for sCNCs.

Assuming the same volume of air injected into the solution, foamability differences can be explained by the higher hydrophobicity of cCNCs. In particle-stabilised foams, foamability depends on the number of particles, the size of aggregates and their hydrophobicity [38]. Interestingly, the polydispersity of cCNC solution is lower than sCNCs, but their interfacial aggregation is much higher, as seen from thin-film balance experiments. The half-life of foams formed in the CNC-LAE suspensions with the double-syringe method is illustrated in Figure 10. The optimal concentration of LAE (0.15 mM) seems to exist for both types of cellulose nanocrystals. Moreover, the lifetime of foam generated in the cCNC suspension was much longer than in the sCNC suspension. In particular, the highly resistant foam could be observed for the suspension of cCNCs and 0.15 mM LAE. That can be explained by the formation of a compact layer of LAE-cCNC aggregates that prevents bubble coarsening and coalescence. Contrary to the thin films’ measurements, where the film lifetime was increased with LAE concentration, the highest foam stability was observed for 0.15 mM LAE for both types of cellulose nanocrystals. This may result from the antifoaming action of larger aggregates that induce premature film rupture [39].

## 4. Conclusions

The suspensions of cellulose nanocrystals (CNCs) with ethyl lauroyl arginate (LAE) have superior foaming properties compared to the counterparts with only individual components, due to the synergistic action at the liquid film interfaces. It is, however, known that the interfacial properties of the suspensions depend on the type of chemical functionality at the surface of cellulose nanocrystals. In present work, the interfacial behaviour of carboxylated cellulose nanocrystals (cCNCs) have been compared with sulfated cellulose nanocrystals (sCNCs) in the presence of LAE. The carboxylated ones (cCNCs) seemed more prone to hydrophobisation with LAE at a concentration much below critical micelle concentration CMC, presumably due to the formation of a bidentate hydrogen bond between the carboxyl groups of cCNCs and guanidine groups of LAE [40]. The drainage experiments in the thin-film balance could exemplify differences between sCNC and cCNC dispersions in the thin films, as it allows direct visualisation of the tendency for particle aggregation and the presence or absence of interfacial flow. The sCNC and cCNC suspensions with LAE formed thin films, with stability increasing with surfactant concentration and with complex rheological properties. While the sCNC aggregates were preferentially present in the film volume with a small fraction at the surface, the cCNC aggregates, due to higher hydrophobicity, were preferentially located at film interfaces, forming compact layers. They also had the tendency to undergo flow-induced aggregation. The presence of both types of aggregates decreased thin liquid film stability compared to the one for the LAE solution with the same concentration.

The LAE solution at concentrations well below CNCs did not foam, and the presence of CNCs in the suspension was critical for its formation. The results of foamability and foam stability are in qualitative agreement with the ones from the thin-film balance experiments. The foam volume increased with the LAE concentration. However, there was an optimum surfactant concentration to achieve a stable foam. In particular, the very resistant foam could be obtained with cCNCs that formed, as the thin-film balance experiments demonstrated, the interfaces with a complex structure and rheology. On the other hand, at high LAE concentrations, the aggregates of CNCs might show antifoaming properties.

Our results indicate that combining the CNC suspension characterisation, thin-film balance analysis and foaming measurements allows the optimisation of LAE-CNC formulations and the determination of the effects affecting foam stability in the complex surfactant/nanoparticle systems. 

## Figures and Tables

**Table 1:** The surface tension of analytical standard, pure and nanoparticle-dispersed ethyl lauroyl arginate. Contents of CNCs, 0.3 wt%.

	Surface Tension [mN/m]		
LAE Concentration [mM]	LAE	LAE–cCNC	LAE–sCNC
0.075	60 ± 0.5	55 ± 1	46 ± 1
0.15	51 ± 0.5	38 ± 1	43 ± 1
0.35	32 ± 0.5	33 ± 1	36 ± 1

**Table 2:** Hydrodynamic diameter and zeta potential of pure cellulose nanocrystals and those in the mixtures with ethyl lauroyl arginate. Contents of CNCs, 0.3 wt%.

	Size [nm] (PDI)	Zeta Potential [mV]	Size [nm] (PDI)	Zeta Potential [mV]
	cCNC		sCNC	
Pure nanocrystals	77 (0.44)	−38 ± 5	96 (0.63)	−44 ± 5
CNC-LAE 0.075 mM	94 (0.40)	−38 ± 5	105 (0.51)	−47 ± 5
CNC-LAE 0.15 mM	75 (0.42)	−33 ± 5	77 (0.52)	−50 ± 5
CNC-LAE 0.35 mM	120 (0.40)	−29.5 ± 5	187 (0.82)	−45 ± 5

## Data Availability

The data presented in this study are openly available from the authors upon reasonable request.

## Supplementary Materials

The following supporting information can be downloaded at: https://www.mdpi.com/article/10.3390/polym14245402/s1, Video S1: LAE 0.075 mM, Video S2: LAE 0.15 mM, Video S3: LAE 0.35 mM, Video S4: LAE 0.075 mM sCNC, Video S5: LAE 0.15 mM sCNC, Video S6: LAE 0.35 mM sCNC, Video S7: LAE 0.075 mM cCNC, Video S8: LAE 0.15 mM cCNC, Video S9: LAE 0.35 mM cCNC.
